# Constructing the Discourse System of “Integrated Care” for Older Adults in China: Logic, Challenges, and Pathways

**DOI:** 10.5334/ijic.10286

**Published:** 2026-04-16

**Authors:** Yue Zheng, Min Liu

**Affiliations:** 1 School of Law, Zhejiang Wanli University, Ningbo, China; 2 Ningbo Research Center for Modernization of Social Governance, Ningbo, China; 3 School of Marxism, Anhui University, Hefei, China

**Keywords:** integrated care, discourse system, elderly care

## Abstract

The construction of an integrated care discourse system for older adults in China significantly influenced the developmental direction of long-term care services, following a “policy-driven and practice-oriented” dual-track logic. National-level policy frameworks establish institutional frameworks, while localized innovations refine service models. Currently, the discourse system confronted four critical challenges: institutional fragmentation between medical and elderly care systems, power imbalance in service provision, policy emphasis on spatial infrastructure over relational integration, and marginalization of service users’ voices. To resolve these barriers, it was imperative to strengthen epistemic subjectivity, established an elderly-centered paradigm, and institutionalize service recipient participation through policy innovation.

## Introduction

The discourse system of integrated care comprises a constellation of discursive practices that articulate its core concepts, implementation strategies, and care models. Based on practical experience, multiple countries have gradually developed models such as the ICCC model for chronic disease care [1], the U.S. PACE program [2], and Canada’s PRISMA project [3].

In China, the concept of “integrated care” did not initially appear explicitly in either academic discourse or practical applications. At the 2009 Ma Yinchu Forum on Population Science, scholars mentioned that community-based care for older adults should consider advocating for the concept of “aging in place”, establishing a continuous care service system, and developing the elderly service industry [4]. This represented an early domestic discussion on what integrated long-term care services should entail and how to achieve them. Subsequently, academia widely recognized the severe fragmentation in elderly policies and service pathways of China [5,6,7]. The term “integration” gradually emerged in domestic academic discourse. Thus, the construction of the integrated care discourse system generally branched into two directions: one actively introducing international experiences, and the other seeking theoretical consciousness for the development of an indigenous knowledge system, such as proposing the construction of a “comprehensive community-based elderly service system” [8] and improving policies for long-term care system of China [9].

Integrated care possesses strong regional characteristics, deeply influenced by local culture, welfare systems, regional environments, and multiple other factors. Constructing an autonomous discourse system of integrated care that can effectively explain and guide local practice is a realistic and urgent task. This paper aims to address this core issue by clarifying the construction logic, identifying practical dilemmas, and exploring development pathways for the “integrated care” discourse system for older adults in China.

### The Logic of Constructing the “Integrated Care” Discourse System for Older Adults

The construction of the “integrated care” discourse system for older adults in China is based on the dual logic of top-level design and grassroots exploration. Top-level design is manifested in national-level strategic planning, policy texts, laws, and regulations, which set the overall development direction and institutional framework for integrated care. Grassroots exploration translates macro policies into specific, operational practice models and summarizes experiences.

In 2013, the State Council issued *Opinions on Promoting the Development of the Health Service Industry*, emphasizing the integration of medical health and elderly care services and encouraging medical institutions to extend nursing services to households. In 2019, the General Office of the State Council issued the *Opinions on Promoting the Development of Elderly Care Services*, which aimed to break down the boundaries among home-based, community-based, and institutional elderly care models.

As of the first half of 2024, there were 87,000 signed cooperation agreements between medical institutions and elderly care institutions nationwide. There were over 7,800 integrated medical and elderly care institutions possessing medical institution qualifications and having completed elderly care institution registration, with a total of 2 million beds [10]. Furthermore, community-embedded senior care homes have developed rapidly in recent years. Taking Shanghai as an example, by the end of 2022, there were 217 care homes for older people [11]. Despite this progress, systemic fragmentation persists, particularly in the form of inter-departmental coordination gaps and challenges in implementing unified policies.

Top-level design and grassroots exploration are not independent and parallel but continuously interact and influence each other. Through this dynamic engagement, the inherent dilemmas in developing an integrated care discourse system have progressively come to light.

### Dilemmas in Constructing the “Integrated Care” Discourse System for Older Adults

#### 1. Institutional Silos Between Medical and Elderly Care

This is specifically manifested in two sets of assessment standards for older adults’ functional capacities that are difficult to interconnect and mutually recognize. The elderly care service system led by the Civil Affairs Department and the long-term care insurance systems led by the Health Commission have formulated different assessment indicators and grading methods based on their respective management logics and policy objectives. This inconsistency in standards not only causes duplication of administrative resources and efficiency losses but, more profoundly, reflects differences in professional services, management philosophies, etc., between the two major systems, creating institutional barriers for advancing integrated care practice.

#### 2. Power Imbalance Between the Healthcare and Long-Term Care Sectors

Within the realm of integrated medical and elderly care services, the healthcare system exerts discursive dominance, asserting predominant authority over critical domains such as basic nursing service standards and infrastructure specifications. For instance, in 2019, the General Office of the National Health Commission issued the *Service Guidelines for Integrated Medical and Elderly Care Institutions (Trial)*, standardizing the setup, service content and requirements, and service processes of integrated institutions. Consequently, when evaluating embedded medical facilities within elderly care institutions, local health supervision departments apply the regulatory standards for hospitals and clinics established by health authorities. Under the medical system’s regulatory framework, elderly care institutions face significant difficulties in embedding their operational characteristics into integrated care management, thereby preventing the formation of a regulatory framework genuinely applicable to the elderly care system.

#### 3. Policy narratives emphasizing spatial infrastructure over relational development

In long-term care policies, common discursive formulations include the “15-minute” home-based elderly care service circle, community-embedded elderly care, and the development of regional elderly care service centers. However, the key element for achieving integrated care lies in the cooperative relationships among service providers within the community space. This requires the integrated care discourse to shift its preference from community space to shaping cooperative relationships among multiple community actors. Furthermore, the essence of integrated care for older adults is being needs-centered. If the discourse system construction overly emphasizes physical environmental changes, it might obscure the essence of integrated care, leading to a disconnect between the discourse and the actual needs of older adults.

#### 4. “Silencing” of Service Users

The user group should be an important stakeholder in constructing the integrated care discourse system but has long been silenced. For instance, in Shanghai, after Long-Term Care Insurance applicants pass the assessment, staff from nursing stations often find it difficult to conduct home visits to comprehensively understand the situation of the older adult and their family. This means that older adults often can only passively receive services and find it hard to actively participate in formulating care plans based on their own needs. Moreover, in the feedback and evaluation stages of long-term care services, older adults and their families are again silenced. Insufficient attention is paid to older adults’ service experiences, unmet needs, or the desired degree of service integration.

### Pathways for Constructing the “Integrated Care” Discourse System for Older Adults

#### 1. Strengthen Epistemic Subjectivity in Constructing the Integrated Care Discourse System

On the one hand, drawing on Chinese practical experiences with integrated care, a comprehensive discourse should be developed that encompasses its core dimensions—including conceptual foundations, sites of practice, strategic actions, service delivery processes, and care outcomes. On the other hand, when incorporating theories and experiences of integrated care from other countries, their contextual applicability must be critically assessed, and key discursive elements should be distilled based on local practices.

#### 2. Establishing a Needs-Centered Philosophy for Discourse System Construction

The concept centered on the needs of older adults is key to breaking down institutional divides between medical and elderly care, balancing power dynamics between the two sectors. In medical-nursing integration practice, inspecting the standardization of services in medical units within elderly care institutions against medical system standards can indeed effectively prevent medical accidents and risks. However, it also overlooks the essential basic medical and nursing needs of older adults. In this regard, pilot programs could be attempted, based on the operational realities of elderly care institutions and centered on meeting the needs of older adults, to formulate a set of practical methods for supervision, management, risk prevention, and resolution in integrated medical and elderly care.

#### 3. Leveraging the Roles of Service Users in Discourse System Construction

Granting service recipients discursive agency is of critical importance, and it is essential to enable them to express themselves fully throughout the care process. To this end, systematically listening to their ongoing articulation of needs and experiences should be regarded as an integral component of care provision and formally incorporated into service delivery workflows.

## Conclusion

Under the backdrop of the comprehensive implementation of the national strategy to proactively address population aging, constructing a discourse system of “integrated care” for the elderly with Chinese characteristics is not only crucial to enhancing the quality and efficiency of long-term care services, but also represents a concrete manifestation of modernizing the national governance system and governance capacity in the field of aging. Currently, although integrated care discourse system of China has initially formed a dual construction logic of “policy-first followed by practical implementation”, it still faces profound challenges in institutional coordination, power structure, narrative focus, and stakeholder participation. Only by rooting in the local context, strengthening the subjectivity awareness in discourse construction, adhering to the authentic needs of the elderly as the core value orientation, and fully activating the discursive power of both service providers and recipients can the development truly transition from “formal integration” to “substantive integration”.
